# The effect of sperm DNA fragmentation on the incidence and origin of whole and segmental chromosomal aneuploidies in human embryos

**DOI:** 10.1530/REP-23-0011

**Published:** 2023-06-23

**Authors:** Jiangman Gao, Zhiqiang Yan, Liying Yan, Xiaohui Zhu, Hui Jiang, Jie Qiao

**Affiliations:** 1 Center for Reproductive Medicine, Department of Obstetrics and Gynecology, Peking University Third Hospital,Peking University Third Hospital, Beijing, China; 2 National Clinical Research Center for Obstetrics and Gynecology (Peking University Third Hospital), Beijing, China; 3 Key Laboratory of Assisted Reproduction (Peking University), Ministry of Education, Beijing, China; 4 Beijing Key Laboratory of Reproductive Endocrinology and Assisted Reproductive Technology (Peking University Third Hospital), Beijing, China

## Abstract

**In brief:**

Whether sperm DNA fragmentation (SDF) affects embryo development and clinical outcomes is still controversial, which limits the utility of SDF testing in assisted reproductive technology management. This study demonstrates that high SDF is associated with the incidence of segmental chromosomal aneuploidy and increased paternal whole chromosomal aneuploidies.

**Abstract:**

We aimed to investigate the correlation of sperm DNA fragmentation (SDF) with the incidence and paternal origin of whole and segmental chromosomal aneuploidies of embryos at the blastocyst stage. A retrospective cohort study was conducted with a total of 174 couples (women aged 35 years or younger) who underwent 238 cycles (including 748 blastocysts) of preimplantation genetic testing for monogenic diseases (PGT-M). All subjects were divided into two groups based on the sperm DNA fragmentation index (DFI) level: low DFI (<27%) and high DFI (≥27%). The rates of euploidy, whole chromosomal aneuploidy, segmental chromosomal aneuploidy, mosaicism, parental origin of aneuploidy, fertilization, cleavage, and blastocyst formation were compared between low- and high-DFI groups. We found no significant differences in fertilization, cleavage, or blastocyst formation between the two groups. Compared to that in the low-DFI group, segmental chromosomal aneuploidy rate was significantly higher in the high-DFI group (11.57% vs 5.83%, *P* = 0.021; OR: 2.32, 95% CI: 1.10–4.89, *P* = 0.028). The whole chromosomal embryonic aneuploidy of paternal origin was significantly higher in cycles with high DFI than in cycles with low DFI (46.43% vs 23.33%, *P* = 0.018; OR: 4.32, 95% CI: 1.06–17.66, *P* = 0.041). However, the segmental chromosomal aneuploidy of paternal origin was not significantly different between the two groups (71.43% vs 78.05%, *P* = 0.615; OR: 1.01, 95% CI: 0.16–6.40,* P* = 0.995). In conclusion, our results suggested that high SDF was associated with the incidence of segmental chromosomal aneuploidy and increased paternal whole chromosomal aneuploidies in embryos.

## Introduction

Chromosome aneuploidy is a frequent event in early human embryos, often leading to poor treatment outcomes from assisted reproductive technology (ART) (Franasiak *et al.* 2014, Tarozzi *et al.* 2021). Embryo aneuploidies are caused by developmental arrest and irregular cell division (Holubcova *et al.* 2015, MacLennan *et al.* 2015). Although most embryonic DNA abnormalities and aneuploidies originate from the oocyte (Hassold & Hunt 2001), the contribution of sperm to embryo aneuploidy remains a topic of intense discussion (Stein *et al.* 2019).

Sperm DNA integrity is crucial to accurately transmitting gene information (Tarozzi *et al.* 2021). The occurrence of breaks in the DNA strand (sperm DNA fragmentation (SDF)) is the most common DNA abnormality in male gametes and might predict male infertility (Agarwal *et al.* 2020, Vaughan *et al.* 2020). Recent evidence suggests that sperm DNA damage is significantly correlated with an increased risk of miscarriage after IVF and intracytoplasmic sperm injection (ICSI) treatment (Tan *et al.* 2019, Dai *et al.* 2021, Haddock *et al.* 2021). The level of SDF in men whose partners have a history of recurrent abortion is higher than that in men with fertile partners (El Hachem *et al.* 2017, McQueen *et al.* 2019). In unexplained recurrent pregnancy loss cases, spermatozoa with aneuploidy, hyperhaploidy, or chromosome 18 disomy are significantly increased (Esquerre-Lamare *et al.* 2018). DNA fragmentation has a significant relationship with sperm aneuploidy (Arumugam *et al.* 2019). Therefore, the higher SDF and probability of sperm aneuploidy can theoretically increase the risk for embryo aneuploidy (Sakkas & Alvarez 2010). However, some previous studies have demonstrated that SDF has no relationship with blastocyst euploidy in IVF cycles (Gat *et al.* 2017, 2018).

In human embryos, most aneuploidies originate from maternal meiotic errors (Rabinowitz *et al.* 2012), which are the leading cause of whole chromosomal aneuploidy (Handyside *et al.* 2012). The rate of whole chromosomal aneuploidy increases with maternal age, making it an obstacle to achieving reproductive success (Franasiak *et al.* 2014). In addition, the incidence of segmental aneuploidies has been reported in oocytes, the first 3 days of embryonic development, and the blastocyst stage (Babariya *et al.* 2017, Kubicek *et al.* 2019). Segmental abnormalities account for 6% of clinical miscarriages (Martinez *et al.* 2010) and affect nearly 0.05% of newborns (Wellesley *et al.* 2012). Distinguishing the parental origin of chromosomal aneuploidies in embryos and confirming the correlation of SDF with embryo aneuploidies of paternal origin is helpful in elucidating the value of SDF in predicting the clinical outcomes of ART treatment.

Next-generation sequencing (NGS) is the most common approach for comprehensive aneuploidy testing by preimplantation genetic testing (PGT) (Rubio *et al.* 2019). A single-nucleotide polymorphism (SNP) array was used to investigate the ploidy state of a single-biopsied blastomere, combined with parental SNP data and crossover frequencies, which can determine the parental origin of aneuploidies inherited by each embryo (Rabinowitz *et al.* 2012). In preimplantation genetic testing of monogenic disease (PGT-M), NGS detects and analyzes embryos after ICSI treatment, selecting embryos with euploid chromosomes and without pathogenic mutation (s) for transfer to the uterus. At the same time, the SNP information of both the parents and the embryos in NGS data allows us to distinguish the parental origin of the aneuploid chromosome (s), including whole chromosomal aneuploidies and segmental chromosomal aneuploidies in embryos, which is comparable with long-read sequencing (Tsuiko *et al.* 2023).

In this study, we aimed to assess the correlation of SDF with the incidence and parental origin of aneuploidies in the blastocyst in the cycles of patients who undergo PGT-M. The primary objective was to evaluate the impact of SDF on the incidence and origin of whole and segmental chromosomal aneuploidies and the secondary objective was to assess and compare fertilization, cleavage, blastocyst formation, and blastocyst formation time.

## Materials and methods

### Subjects

A retrospective cohort study was conducted at our reproductive medicine center from January 2018 to July 2022. The study included cycles involving PGT-M, in which SDF was analyzed. All couples who underwent IVF/PGT-M treatments were evaluated for karyotype abnormalities, and SDF was tested according to the patient's wishes prior to IVF/PGT-M treatment. Patients with karyotype abnormalities were excluded. This study was approved by the Institutional Review Board of Peking University Third Hospital (reference no: 2017SZ-028). Cycles with a maternal age ≤35 years were included to reduce the influence of female factors on embryo aneuploidy, since there is general consensus on the definition advanced maternal age, which is over 35 years (Lean *et al.* 2017). Then, the cycles were divided into two groups according to the sperm DNA fragmentation index (DFI): low DFI (<27%) and high DFI (≥27%).

### Semen analysis

Semen samples were collected after 2–5 days of abstinence. After liquefaction, the sperm concentration and progressive motility were assessed by a computer-assisted sperm analysis system (SSA-II, Suijia Software Co. Ltd., Beijing, China). Normal sperm morphology was evaluated in samples stained under Papanicolaou staining. SDF was tested using a sperm chromatin structure assay (SCSA) kit. Determination of the sperm DFI (%) was performed according to the method described in detail by Evenson *et al.* (Evenson *et al.* 2020).

### Ovarian reserve evaluation

Blood samples from women were collected on days 2–4 of menstruation to assess the baseline levels of FSH, estradiol (E_2_), and anti-Müllerian hormone (AMH). The antral follicle count (AFC) was determined by transvaginal ultrasound.

### Ovarian stimulation, laboratory protocols, and definition

Controlled ovarian hyperstimulation, oocyte retrieval, fertilization, and culture protocols were performed according to routine clinical and laboratory procedures (Lin *et al.* 2017). The fertilization rate was calculated as the ratio of fertilized oocytes to the number of eggs undergoing ICSI per cycle. The cleavage rate was calculated as the number of day-3 embryos divided by the number of fertilized oocytes. The embryos were incubated in an incubator 5–7 days after ICSI, and blastocyst biopsy was performed after blastocyst formation. The blastulation rate was defined as the number of blastocysts suitable for trophectoderm (TE) biopsy divided by the number of fertilized oocytes. The euploidy rate was the number of blastocysts with normal karyotypes divided by the number of biopsied and successfully analyzed blastocysts. If at least one whole chromosomal aneuploidy was detected in the TE biopsy, the blastocyst was defined as whole aneuploidy. The blastocysts of segmental aneuploidy and mosaicism were defined in the same way. The aneuploidy and mosaicism rates were calculated as the number of aneuploid and mosaic blastocysts divided by the number of blastocysts successfully analyzed.

### Blastocyst biopsy and cryopreservation

On day 3 after ICSI, the zona pellucida was drilled by a laser. TE cells protruded from the holes. All the cycles were subjected to TE-cell biopsy with a laser. A total of 3–10 TE cells were aspirated on day 5, day 6, or day 7 with a biopsy needle under laser assistance. Blastocysts were vitrified individually and stored in liquid nitrogen after being biopsied. The TE cells were rinsed two times with phosphate-buffered saline containing 0.1% human serum albumin (HSA) (Irvine Scientific, Irvine, CA, USA) and then quickly transferred to a 0.2-mL PCR tube with 5 μL lysis buffer.

### Whole-genome amplification and NGS library construction

The whole genomes of the biopsied cells were amplified by multiple annealing and looping-based amplification cycles (MALBAC) according to previous studies (Ren *et al.* 2016, Yan *et al.* 2018). The main steps were as follows. For each reaction, 5 μL of the sample and 30 μL of preamplification agent were combined. Then, each reaction was incubated at 94°C for 3 min for one cycle; amplified at 20°C for 40 s, 30°C for 40 s, 40°C for 30 s, 50°C for 30 s, 60°C for 30 s, 70°C for 4 min, 20 s at 95°C, and 58°C for 10 s for eight cycles; and stopped at 4°C. Subsequently, each reaction system was supplemented with 30 μL of amplification reaction mix. Then, the reactions were incubated at 94°C for 3 min; amplified at 94°C for 20 s, 58°C for 30 s, and 72°C for 3 min for 17 cycles; and stopped at 4°C. The amplified products were stored at −20°C. The amplified embryonic DNA and pedigree DNAs were purified and broken into fragments with an average size of 300 bp by the Covaris M2 system. The NGS library was constructed with the library construction kit. The constructed NGS library was sequenced in a 150-bp paired-end model with an Illumina HiSeq Xten Sequencer (Illumina, USA).

### Sequencing data analysis

The adapter, low-quality base, and MALBAC primer were removed from the raw data, and reads larger than 36 bp were retained. The processed reads were aligned to the human reference genome (hg38) to generate bam files. The reads with a MAPQ lower than 1 and duplicated reads introduced in the PCR were removed. Embryonic copy number variation analysis was conducted according to a previous study (Yan *et al.* 2018). Mosaicism is characterized by the simultaneous existence of two or more genetically different cell lines in an embryo. The level of mosaicism was calculated by the copy number ratio and reported with a range of 20–80% mosaic level. Mosaic levels ≤20% was defined as euploidy mosaic levels >80% was defined as aneuploidy. Segmental aneuploidy was determined when a fragment of a chromosome >10 Mb in size lost or gained deviated from the standard thresholds for euploidy. To identify the parental origin of each aneuploid chromosome in the embryo, we separated the reads covering the parental-distinguished SNP sites in the embryo genome, and the parental origin of aneuploidy was identified by our previously described method (Wang *et al.* 2021).

### Statistical analysis

Statistical analyses were performed using SPSS 26.0 software (IBM Corp.). Continuous data are expressed as the mean ± standard error (s.e.), and categorical variables are expressed as counts (percentages). Numerical variables were evaluated using the Mann‒Whitney *U*-test. Categorical variables were determined using the chi-squared test. In the multivariate logistic regression analysis, female age, male age, AMH, AFC, sperm concentration, progressive motility, and DFI were included, and the data are reported as odds ratios (ORs) and 95% confidence intervals (95% CIs). All tests were two-sided, and a *P-*value <0.05 was considered statistically significant.

## Results

A total of 174 couples who underwent PGT-M were included, and 238 cycles were performed. The average age of the female subjects was 30.94 ± 0.19 years, and the average age of the male subjects was 32.68 ± 0.25 years. A total of 3614 oocytes, including 2813 (77.84%) metaphase II (MII) oocytes, were obtained. Among these MII oocytes, 2228 (79.20%) oocytes were successfully fertilized by ICSI. A total of 1828 (82.05%) day-3 embryos and 748 (33.57%) blastocysts were obtained. The percentages of day-5, day-6, and day-7 blastocysts were 19.79%, 77.27%, and 2.94%, respectively. From these blastocysts, more than 98% (739/748) of TE biopsies were successfully analyzed. A total of 512 (69.28%) blastocysts were diagnosed with normal karyotypes (euploidy). At least one whole chromosomal aneuploidy was detected in 101 (13.67%) TE biopsies, whereas at least one segmental aneuploidy was detected in 50 (6.77%) TE biopsies. There were four (0.54%) samples that had both whole chromosomal aneuploidy and segmental chromosomal aneuploidy. Mosaicism was detected in 89 (12.04%) blastocysts (Supplementary Table 1, see section on supplementary materials given at the end of this article).

First, we compared the cycle characteristics and embryo development of the sperm DFI < 27% and DFI ≥ 27% groups, which included 143 patients underwent 194 cycles and 31 patients underwent 44 cycles, respectively. Maternal age and paternal age were significantly higher in the DFI ≥ 27% group than in the DFI < 27% group (32.14 ± 0.41 vs 30.66 ± 0.21, *P* = 0.002; 34.82 ± 0.76 vs 32.20 ± 0.25, *P* < 0.001, respectively). The progressive motility of sperm was significantly lower in the DFI ≥ 27% group than in the DFI < 27% group (17.98 ± 2.00 vs 32.70 ± 1.28, *P* < 0.001). However, there were no significant differences in fertilization, cleavage, blastulation, or blastocyst formation time between the two groups (Table 1).

**Table 1:** Comparison of cycle characteristics and embryo development between two groups of sperm DFI. Continuous data are expressed as the mean ± s.e., and categorical variables are expressed as *n* (%); numerical variables were evaluated using the Mann–Whitney *U-*test. Categorical variables were determined using the chi-squared test. A *P*-value <0.05 indicated a statistically significant difference.

Variable	DFI		*P*-value
	<27%	≥27%	
Patients (*n*)	143	31	
Cycles (*n*)	194	44	
Maternal age (years)	30.66 ± 0.21	32.14 ± 0.41	**0.002**
Paternal age (years)	32.20 ± 0.25	34.82 ± 0.76	**<0.001**
Basal FSH (mIU/mL)	6.72 ± 0.13	6.73 ± 0.31	0.677
E_2_ (pmol/L)	152.69 ± 3.82	169.98 ± 5.98	0.063
AMH (ng/mL)	3.78 ± 0.23	3.53 ± 0.42	0.830
AFC (*n*)	16.51 ± 0.6	17.16 ± 1.03	0.279
Sperm concentration (10^6^/mL)	69.13 ± 3.33	60.24 ± 5.74	0.295
Progressive motility (%)	32.70 ± 1.28	17.98 ± 2.00	**<0.001**
Normal morphology (%)	3.04 ± 0.11	2.64 ± 0.16	0.468
Retrieved oocyte (*n*)	15.25 ± 0.53	14.89 ± 1.13	0.860
Mature oocyte rate (%)	78.48 ± 1.16	79.75 ± 2.40	0.608
Fertilization rate (%)	78.77 ± 1.18	81.78 ± 2.26	0.280
Cleavage rate (%)	81.13 ± 1.40	84.52 ± 2.71	0.250
Blastulation rate (%)	34.02 ± 1.58	32.92 ± 3.19	0.563
Day 5	127 (20.32)	21 (17.07)	0.296
Day 6	482 (77.12)	96 (78.05)	
Day 7	16 (2.56)	6 (4.88)	

AFC, antral follicle count; AMH, anti-Müllerian hormone; DFI, DNA fragmentation index; E2, Estradiol; FSH, Follicle stimulating hormone_._

**Figure 1:**
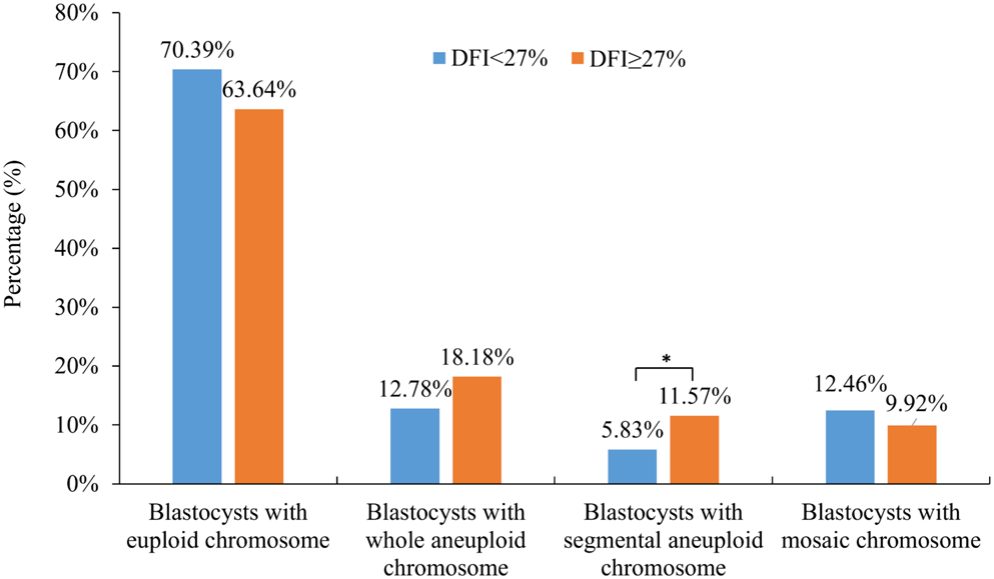
The proportions of blastocysts with euploidy and chromosomal abnormalities between the DFI < 27% and DFI ≥ 27% groups. Data were presented as a percentage. The proportions between the two groups were tested by the chi-square test. *P = 0.021.

The proportions of blastocysts with euploidy and chromosomal abnormalities between the DFI < 27% and DFI ≥ 27% groups are presented in Fig. 1 and Supplementary Table 2. No significant differences in euploidy rates (70.39% and 63.64%) or mosaicism rates (12.46% and 9.92%) were found between the two groups. The whole aneuploidy rate in the DFI ≥ 27% group was higher than that in the DFI < 27% group (12.78% and 18.18%), although the difference was not significant. However, the number of blastocysts with segmental aneuploid chromosomes was significantly higher in the DFI ≥ 27% group than in the DFI < 27% group (11.57% vs 5.83%, *P* = 0.021). Logistic regression analysis showed that sperm DFI level was significantly associated with segmental aneuploidy in blastocysts (*P* = 0.028, OR: 2.32, 95% CI: 1.10–4.89) but not with euploidy (*P* = 0.250, OR: 0.76, 95% CI: 0.47–1.22), whole chromosomal aneuploidy (*P* = 0.434, OR: 1.30, 95% CI: 0.68–2.49) or mosaicism (*P* = 0.819, OR: 0.92, 95% CI: 0.46–1.84) (Supplementary Table 3).

We further analyzed the parental origin of the whole aneuploidy and segmental aneuploidy. The whole chromosome aneuploidies of paternal origin increased significantly in the DFI ≥ 27% group compared with the DFI < 27% group (46.43% vs 23.33%, *P* = 0.018). However, the parental origin of segmental aneuploidy was not significantly different between the two groups (71.43% vs 78.05%, *P* = 0.615) (Table 2). Logistic regression analysis showed that maternal age and sperm DFI were significantly associated with whole chromosome aneuploidies of paternal origin (*P* = 0.049, OR: 0.77, 95% CI: 0.60–1.00; *P* = 0.041, OR: 4.32, 95% CI: 1.06–17.66, respectively) but not associated with segmental chromosome aneuploidies of paternal origin (*P* = 0.329, OR: 1.22, 95% CI: 0.82–1.80; *P* = 0.995, OR: 1.01, 95% CI: 0.16– 6.40, respectively) (Table 3).

**Table 2:** Parental origins aneuploid chromosome related to sperm DFI. Data are presented as *n* (%).

Variable	Parental origin of whole aneuploidy			Parental origin of segmental aneuploidy		
	Maternal	Paternal	*P*-value	Maternal	Paternal	*P*-value
DFI < 27%	69 (76.67)	21 (23.33)	**0.018**	9 (21.95)	32 (78.05)	0.615
DFI ≥ 27%	15 (53.57)	13 (46.43)		4 (28.57)	10 (71.43)

**Table 3:** Logistic regression analysis of the paternal origin of chromosomal aneuploidy.

Variables	Whole chromosomal aneuploidy		Segmental chromosomal aneuploidy	
	*P*-value	OR (95% CI)	*P*-value	OR (95% CI)
Maternal age	**0.049**	0.77 (0.60–1.00)	0.329	1.22 (0.82–1.80)
Paternal age	0.414	1.12 (0.85–1.48)	0.620	0.91 (0.63–1.32)
AMH	0.689	0.95 (0.75–1.21)	0.090	0.72 (0.49–1.05)
AFC	0.381	1.05 (0.95–1.15)	0.943	1.00 (0.86–1.15)
Sperm concentration	0.113	1.01 (1.00–1.03)	0.935	1.00 (0.98–1.02)
Progressive motility	0.792	1.00 (0.97–1.04)	0.135	1.05 (0.99–1.12)
DFI < 27%		Ref		Ref
DFI ≥ 27%	**0.041**	4.32 (1.06–17.66)	0.995	1.01 (0.16–6.40)

AFC, antral follicle count; AMH, anti-Müllerian hormone; CI, confidence interval; DFI, DNA fragmentation index; OR, odds ratio; Ref, reference group.

## Discussion

In the present study, we aimed to explore the correlations between SDF and fertilization, cleavage, blastocyst formation, euploidy and chromosomal abnormalities after ICSI-PGT-M treatment in couples with monogenic diseases and to analyze the relationship between SDF and the incidence and origin of whole and segmental chromosomal aneuploidies in human embryos. The results showed that a higher DFI in sperm was not significantly correlated with the fertilization rate, cleavage rate, or blastocyst formation rate. Euploidy, whole chromosomal aneuploidy and mosaicism were not affected by SDF. However, the segmental chromosomal aneuploidy and paternal origin of whole chromosomal aneuploidy were significantly higher in the DFI ≥ 27% group. To the best of our knowledge, this is the first study to investigate the relationship between SDF and the parental origins of whole and segmental chromosomal aneuploidy in embryos.

The question of whether SDF affects embryo development and clinical outcomes is still controversial, which limits the utility of SDF testing in ART management. Some previous studies demonstrated that a high level of SDF is related to diminished fertilization and embryo development and a decreased pregnancy rate (Benchaib *et al.* 2003, Benchaib *et al.* 2007, Oleszczuk *et al.* 2016, Xue *et al.* 2016, Alvarez Sedo *et al.* 2017). However, some studies reported that SDF does not affect the fertilization rate, embryo quality, clinical pregnancy rate, or miscarriage rate (Gandini *et al.* 2004, Anifandis *et al.* 2015, Esbert *et al.* 2018, Sun *et al.* 2018, Yang *et al.* 2019, Green *et al.* 2020). To exclude the influence of female factors, Antonouli *et al.* studied the relationship between DFI and embryo development or clinical outcome in an egg-recipient population. The results showed that DFI is positively correlated with male age and negatively correlated with total sperm count and progressive motility but not significantly correlated with pregnancy outcome (Antonouli *et al.* 2019). A study by Esbert *et al.* also suggested that SDF did not affect fertilization, embryo quality, clinical pregnancy, or miscarriage in IVF cycles with own or donor eggs (Esbert *et al.* 2011). Green *et al.* suggested that there was a similar rate of blastulation in couples with low and high DNA fragmentation (Green *et al.* 2020). In our study, patients with a maternal age of more than 36 years were excluded to control for the effect of female age on the aneuploidy of embryos. The results showed that sperm DFI had no significant correlation with fertilization, cleavage, or blastulation, which was consistent with most previous studies.

Embryonic aneuploidy is considered the main contributor to implantation failure (Scott *et al.* 2012, Franasiak *et al.* 2014), especially in cases of recurrent pregnancy loss (Jia *et al.* 2015, Sato *et al.* 2019). Most aneuploidies in embryos are of maternal origin, and the incidence of aneuploidy varies with the age of the woman. The rate of aneuploidy in women aged 23 years and under is 40% and that in women aged 26–30 years is the lowest, at approximately 20–27%. The incidence of aneuploidy increases gradually from 31 to 43 years of age, reaching a high plateau of approximately 85% (Franasiak *et al.* 2014). Aging reduces the ovarian reserve and increases the rate of oocyte aneuploidy, which may be due to meiotic errors in oocytes (Rodrigo 2020). In this study, logistic regression analysis showed that whole chromosomal aneuploidy increased significantly with maternal age, although female age was controlled. However, the paternal whole chromosomal aneuploidy and segmental chromosomal aneuploidy in embryo did not increase with maternal age In order to minimize the influence of female age on embryonic aneuploidy, their age was controlled to be not above 35 years in this study.

Although the effect of SDF on embryo development and the clinical outcomes after IVF/ICSI remain controversial, some studies have demonstrated that SDF is related to sporadic miscarriage (Robinson *et al.* 2012) and recurrent pregnancy loss (McQueen *et al.* 2019, Tan *et al.* 2019). The clinical practice guidelines recommend SDF testing for idiopathic male infertility, unexplained male infertility, and recurrent pregnancy loss (grade C recommendation) (Agarwal *et al.* 2020). Embryonic aneuploidy is considered the main contributor to implantation failure (Scott *et al.* 2012, Franasiak *et al.* 2014), especially in cases of recurrent pregnancy loss (Jia *et al.* 2015, Sato *et al.* 2019). Male factors also seem to contribute to embryo aneuploidy and infertility (Rodrigo *et al.* 2019). High sperm aneuploidy levels are inversely associated with clinical outcomes in infertile couples undergoing ICSI cycles (Rubio *et al.* 2001, Burrello *et al.* 2003, Nicopoullos *et al.* 2008). A significant positive relationship was observed between SDF and sperm aneuploidy (Enciso *et al.* 2013, Arumugam *et al.* 2019). As stated above, DNA fragmentation in spermatozoa is likely to be associated with embryo aneuploidy. However, previous studies and our evidence show that there is no significant correlation between SDF and blastocyst whole chromosomal aneuploidy (Bronet *et al.* 2012, Gat *et al.* 2017, 2018, Green *et al.* 2020). Nevertheless, whether high SDF increases the risk of abnormalities of paternal chromosomes in aneuploid embryos is unknown. If we determine the association between SDF and paternal chromosomal abnormalities, we can understand the effect of sperm DNA damage on embryo development more clearly. The analysis for the parental origin of aneuploidies showed that the whole chromosomal aneuploidies of paternal origin was significantly higher in the high-DFI group. This suggests that severe DNA damage in spermatozoa contributes to the occurrence of whole chromosomal aneuploidies in embryos.

In addition to whole chromosomal aneuploidies, *de novo* segmental chromosomal aneuploidies arising from chromosomal structural rearrangements are relatively common in embryos (Babariya *et al.* 2017). The incidence of segmental aneuploidies at the blastocyst stage ranges from 5.1% to 8.4% (Escriba *et al.* 2019, Kubicek *et al.* 2019, Girardi *et al.* 2020). Segmental aneuploidies more frequently affect paternal chromosomes, whereas whole chromosomal aneuploidies more often involve maternal chromosomes in aneuploid embryos (Kubicek *et al.* 2019). In our present study, 28.8% of whole aneuploid chromosomes were of paternal origin, while 76.4% of segmental aneuploidies affected paternal chromosomes. Kubicek *et al.* speculated that SDF might be a reason why most of the segmental changes affect paternal chromosomes. In this study, segmental chromosomal aneuploidy was significantly higher when the DFI was ≥27%. However, the parental origin of segmental aneuploidies was not significantly different between the DFI < 27% group and the DFI ≥ 27% group. Paternal segmental aneuploidy in the embryos may be related to other impaired DNA integrity in sperm, such as defective maturation (a lack of full exchange of histones for protamines) and Fas-mediated abortive apoptosis. Further studies are needed to establish a clear understanding of segmental aneuploidy in embryos.

There are some limitations in our study. First, this observational study had some limitations associated with its retrospective nature: uncontrolled biases and incomplete data such as smoking and drugs. Second, sperm DFI was detected around the day of egg retrieval not the exact day for ICSI. Third, although the PGT-M patients did not have an infertility problem, and PGT-M did not increase the risk of aneuploidy in their embryos, the sample size of PGD-M patients was too small, so further prospective study with larger cohorts is necessary to confirm the present findings.

In conclusion, this study demonstrates that SDF is not associated with fertilization, cleavage, or blastulation after ICSI treatment. High levels of SDF are probably associated with higher segmental chromosomal aneuploidies and potential paternal whole chromosomal aneuploidies in embryos. Despite the novelty of this study, it had some limitations.

## Supplementary Materials

Supplemental table 1. Description of the study population and embryos.

Supplemental table 2 The blastocysts with euploidy and chromosomal abnormalities between the DFI<27% and DFI≥27% groups.

Supplementary table 3 Logistic regression analysis of the chromosomal euploidy, aneuploidy and mosaicism of blastocysts.

## Declaration of interest

The authors declare that the research was conducted in the absence of any commercial or financial relationships that could be construed as a potential conflict of interest.

## Funding

This work was supported by the National Key Research and Development Program of China (2017YFA0103801, 2019YFA0801400 and 2021YFC2700203), the Beijing Municipal Science & Technology Commission (Z191100006619075), and the National Natural Science Foundation of China (81871204 and 81901535).

## Author contribution statement

J Qiao and LYan planned and designed the study. J Gao and X Zhu were responsible for the data collection. Z Yan conducted the embryo aneuploidy analysis, while J Gao, L Yan, and H Jiang contributed to the analysis and interpretation of the results. J Gao drafted the article, while all authors critically revised the manuscript and approved the final version.
