# Supplementary material for: The effect of sperm DNA fragmentation on the incidence and origin of whole and segmental chromosomal aneuploidies in human embryos
Source: Reproduction. 2023 Jun 23;166(2):117–24. doi: 10.1530/REP-23-0011 (PMC10326632; doi:10.1530/REP-23-0011)
Supplement: Supplemental table 1. Description of the study population and embryos. [file supplementary_table_1.pdf]

Supplemental table 1. Description of the study population and embryos.

Variable	Description
Patient (n)	174
Number of cycles (n)	238
Female age (y)	30.94±0.19
Male age (y)	32.68±0.25
Basal FSH (mIU/ml)	6.72±0.12
E_2 (pmol/L)	154.04±3.31
AMH (ng/ml)	3.73±0.20
AFC (n)	16.63±0.52
Sperm concentration (10^6/ml)	67.48±2.92
Progressive motility (%)	29.98±1.17
Normal morphology (%)	2.96±0.09
Number of retrieved oocytes (n)	3614
Mature oocytes (n, %)	2813 (77.84)
Number of fertilized embryos (n, %)	2228 (79.20)
Cleavage embryos (n, %)	1828 (82.05)
Blastocysts development (n, %)	748 (33.57)
Day 5 (n, %)	148 (19.79)
Day 6 (n, %)	578 (77.27)
Day 7 (n, %)	22 (2.94)
Blastocysts with genetic results (n, %)	739 (98.80)
Euploid blastocysts (n, %)	512 (69.28)
Whole chromosomal aneuploid blastocysts (n, %)	101 (13.67)
Segmental chromosomal aneuploid blastocysts (n, %)	50 (6.77)
Mosaic blastocysts (n, %)	89 (12.04)
Whole aneuploid chromosomes (n)	118
Maternal origin (n, %)	84 (71.19)
Paternal origin (n, %)	34 (28.81)
Segmental aneuploid chromosomes (n)	55
Maternal origin (n, %)	13 (23.64)
Paternal origin (n, %)	42 (76.36)

Note: Continuous data are expressed as the mean ± standard error (SE), and categorical variables are expressed as counts (percentages); DFI, DNA fragmentation index; FSH, Follicle stimulating hormone; E_2, Estradiol; AMH, anti-Müllerian hormone; AFC, Antral follicle count.
