# Supplementary material for: The effect of sperm DNA fragmentation on the incidence and origin of whole and segmental chromosomal aneuploidies in human embryos
Source: Reproduction. 2023 Jun 23;166(2):117–24. doi: 10.1530/REP-23-0011 (PMC10326632; doi:10.1530/REP-23-0011)
Supplement: Supplemental table 2 The blastocysts with euploidy and chromosomal abnormalities between the DFI<27% and DFI≥27% groups. [file supplementary_table_2.pdf]

Supplemental table 2 The blastocysts with euploidy and chromosomal abnormalities between the DFI<27% and DFI≥27% groups.

Group	Total (n)	Euploidy blastocysts (n, %)	Whole aneuploidy blastocysts (n, %)	Segmental aneuploidy blastocysts (n, %)	Mosaic blastocysts (n, %)
DFI <27%	618	435(70.39%)	79 (12.78%)	36 (5.83%)	77 (12.46%)
DFI⩾27%	121	77(63.64%)	22 (18.18%)	14 (11.57%)*	12 (9.92%)

Note: The chi-square test was used. * *P*=0.021
