# Supplementary material for: The effect of sperm DNA fragmentation on the incidence and origin of whole and segmental chromosomal aneuploidies in human embryos
Source: Reproduction. 2023 Jun 23;166(2):117–24. doi: 10.1530/REP-23-0011 (PMC10326632; doi:10.1530/REP-23-0011)
Supplement: Supplementary table 3 Logistic regression analysis of the chromosomal euploidy, aneuploidy and mosaicism of blastocysts. [file supplementary_table_3.pdf]

Supplementary table 3 Logistic regression analysis of the chromosomal euploidy, aneuploidy and mosaicism of blastocysts.

Variables	Euploid blastocysts		Whole chromosomal aneuploid blastocysts		Segmental chromosomal aneuploid blastocysts		Mosaic chromosomal blastocysts	
	P value	OR (95% CI)	*P* value	OR (95% CI)	*P* value	OR (95% CI)	*P* value	OR (95% CI)
Maternal age	0.393	0.96 (0.88-1.05)	**0.031**	1.15 (1.10-1.32)	0.913	0.99 (0.84-1.17)	0.432	0.95 (0.84-1.08)
Paternal age	0.170	1.06 (0.98-1.15)	0.122	0.91 (0.81-1.03)	0.754	0.98 (0.84-1.13)	0.746	0.98 (0.88-1.10)
AMH	0.243	1.05 (0.97-1.15)	0.344	0.95 (0.84-1.06)	0.115	0.88 (0.74-1.03)	0.545	1.04 (0.93-1.16)
AFC	0.404	0.99 (0.96-1.02)	0.715	0.99 (0.95-1.04)	0.067	1.05 (1.00-1.11)	0.863	1.00 (0.96-1.04)
Sperm concentration	0.203	1.00 (1.00-1.01)	0.117	1.00 (0.99-1.00)	0.674	1.00 (0.99-1.01)	0.617	1.00 (0.99-1.00)
Progressive motility	0.730	1.00 (0.99-1.01)	0.126	1.01 (1.00-1.03)	0.570	1.01 (0.99-1.03)	0.158	0.99 (0.97-1.00)
DFI<27%		Ref		Ref		Ref		Ref
DFI≥27%	0.250	0.76 (0.47-1.22)	0.434	1.30 (0.68-2.49)	**0.028**	2.32 (1.10-4.89)	0.819	0.92 (0.46-1.84)

Note: Ref, reference group; OR, odds ratios; CI, confidence intervals; DFI, DNA fragmentation index; FSH, Follicle stimulating hormone; E_2, Estradiol; AMH, anti-Müllerian hormone; AFC, Antral follicle count.
